# Topographical Patterns of Intraventricular Hemorrhage as a Novel Prognostic Imaging Biomarker in Intracerebral Hemorrhage

**DOI:** 10.1002/cns.71071

**Published:** 2026-08-10

**Authors:** Ao Chen, Xufan Yu, RenHui Zhou, Xun Chen, JianXian Li, JiaYing Peng

**Affiliations:** ^1^ Department of Neurosurgery YueYang People's Hospital Yueyang Hunan China; ^2^ School of Intelligent Transportation Hunan Communication Engineering Polytechnic Changsha Hunan China; ^3^ Department of Neurosurgery The Fourth People's Hospital of Changsha Changsha Hunan China; ^4^ Department of Infectious Diseases YueYang People's Hospital Yueyang China

**Keywords:** intracerebral hemorrhage, intraventricular hemorrhage, prognostic model, topographical patterns

## Abstract

**Aims:**

Current intraventricular hemorrhage (IVH) scoring systems quantify individual hemorrhage site features but do not account for differences in overall IVH distribution. We aimed to develop a scoring system based on overall IVH topographical patterns, and evaluate its value for predicting outcomes in intracerebral hemorrhage (ICH).

**Methods:**

We conducted a retrospective analysis of a database including 385 patients with spontaneous ICH, IVH, and hematoma volumes < 40 mL between June 2019 and October 2024. We identified independent predictors of 30‐day mortality, evaluated whether IVH spatial features could replace existing IVH scores, developed a novel IVH topographical score (ITS) scoring system, and compared it with four established scoring systems.

**Results:**

The IVH topographical patterns demonstrated significantly superior predictive performance for mortality compared with the original and modified Graeb scores. Multivariate analysis identified IVH topographical patterns, hydrocephalus, hematoma volume, and Glasgow Coma Scale score as independent predictors. The ITS demonstrated superior predictive performance for 30‐day mortality compared with the other four, including in the external validation cohort (*n* = 102), while its performance for 6‐month outcomes was comparable to the other four.

**Conclusion:**

Topographical patterns of IVH provide an intuitive tool for risk stratification in patients with IVH. However, larger prospective studies are warranted to validate these findings in broader populations.

## Graphical abstract

This study developed and externally validated the IVH topographical score (ITS) based on 11 IVH topographical patterns and clinical variables. The ITS classifies IVH patterns into four risk levels (0–3 points) and integrates GCS (3–8: 1 point), ICH volume (≥ 20 mL: 1 point), and hydrocephalus (4 points). The total score (0–9) stratifies patients into low (0–2), intermediate (3–6), and high risk (7–9) groups. The ITS serves as a simple and effective tool for bedside risk stratification in patients with IVH.

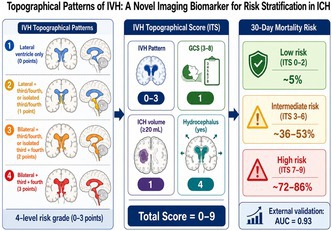

**Abbreviations**
AUC: area under the receiver operating characteristic curve
CT: computed tomography
EVD: external ventricular drain
GCS: Glasgow Coma Scale
ICH: intracerebral hemorrhage
ITS: IVH topographical score
IVH: intraventricular hemorrhage
mGS: modified Graeb score
mICH: A and mICH‐B, modified ICH scores
mRS: modified Rankin Scale
oGS: original Graeb score
oICH: original ICH score
ROC: receiver operating characteristic

## 1 Introduction

Intracerebral hemorrhage (ICH) is the most severe type of stroke, accounting for 10%–15% of all cases [1]. Intraventricular hemorrhage (IVH) occurs in 30%–45% of patients with ICH [2, 3, 4]. Multiple studies have confirmed that IVH is an independent risk factor for poor prognosis in patients with ICH and significantly increases 30‐day mortality risk [5, 6, 7, 8, 9].

Although several ICH prognostic scoring systems have been developed, only a subset considers the impact of IVH, and most treat it as a binary variable (present or absent), limiting their ability to assess outcomes in patients with IVH [10, 11, 12, 13]. To specifically evaluate IVH severity, tools such as the IVH [14], Graeb [15], and Le Roux [16] scores have been developed. These scoring systems primarily assess severity by quantifying total hemorrhage volume and ventricular dilatation, but typically lack the ability to distinguish specific ventricular regions, which may restrict their correlation with true IVH volume and limit predictive performance.

In 2013, Morgan et al. [17] modified the original Graeb score (oGS) using data from the CLEAR B trial, improving its correlation with actual hematoma volume changes and clinical outcomes by incorporating detailed assessment of hemorrhage in specific ventricular locations. Despite such refinements, existing IVH scoring systems still lack direct prognostic value for patients with ICH and remain relatively complex and time‐consuming, limiting their widespread applicability in clinical practice. Therefore, a reliable, simple, intuitive, and clinically meaningful tool for IVH assessment is urgently needed. Although previous studies have linked hemorrhage across different ventricular locations with poor outcomes [9, 18, 19, 20], the prognostic impact of overall IVH distribution patterns and their corresponding risks have not been systematically quantified.

We hypothesized that the overall topographical distribution characteristics of IVH could serve as a novel imaging biomarker for predicting poor prognosis and that associated risk levels could be quantified, replacing existing IVH scoring systems to directly predict outcomes in patients with IVH. Therefore, we developed a novel scoring system based on the different overall IVH distribution characteristics in a retrospectively analyzed cohort and validated its predictive performance in an external cohort.

## 2 Methods

### 2.1 Study Design and Population

The internal cohort was derived from a prospectively established standardized clinical database of patients with spontaneous ICH at the Stroke Center of Yueyang People's Hospital between January 2019 and October 2024. For external validation, an independent cohort from a prospectively maintained registry at the Stroke Center of the Fourth People's Hospital of Changsha between December 2024 and June 2025 was used. Both cohorts strictly adhered to the same eligibility criteria.

Inclusion criteria were as follows: (1) computed tomography (CT)‐confirmed ICH with ventricular extension or primary IVH; (2) hematoma volume < 40 mL; (3) 18–90 years of age; and (4) hospital admission and treatment initiation within 24 h of ICH onset. Exclusion criteria were as follows: (1) IVH secondary to traumatic brain injury, ruptured aneurysm or vascular malformation, spontaneous subarachnoid hemorrhage, tumor hemorrhage, or ischemic stroke; (2) brainstem hemorrhage; and (3) severe physical or psychiatric conditions causing significant functional impairment or life‐threatening complications.

All patients were diagnosed and treated according to the Chinese Guidelines for Diagnosis and Treatment of Intracerebral Hemorrhage (2019) [21]. Surgical hematoma evacuation was generally considered for supratentorial primary hemorrhage ≥ 30 mL or cerebellar hemorrhage ≥ 10 mL with reduced consciousness. External ventricular drain (EVD) placement was performed in patients with hydrocephalus, with bilateral EVD placement when CT showed complete obstruction of the third ventricle or bilateral occlusion of the foramina of Monro. All EVDs were placed within 24 h of repeat cranial CT. EVD removal was considered when the daily drainage volume was < 100 mL and no progressive ventricular enlargement or elevated intracranial pressure was observed 48 h after clamping.

### 2.2 Baseline Characteristics

Demographic, clinical, imaging, and treatment data were collected from patient medical records, including sex, age, medical history, level of consciousness on admission, body temperature, systolic and diastolic blood pressure, hemorrhage location, hematoma volume, treatment modality, IVH scores, and overall IVH topographical characteristics.

### 2.3 Radiological Assessment

Initial CT scans were performed within 24 h of IVH onset and prior to EVD placement. Parenchymal hematoma volume was calculated using the formula (1/2 × A × B × C), where A, B, and C represented the maximum measured width, length, and height, respectively. IVH severity was evaluated according to the oGS (range, 0–12) and modified Graeb score (mGS; range, 0–32). Hydrocephalus was diagnosed on the initial CT scan obtained at admission, using the following criteria: maximum frontal horn diameter divided by the maximum cranial diameter in that plane (Evans' index) > 0.3, maximum frontal horn diameter > 45 mm, third ventricular diameter > 6 mm, or fourth ventricular diameter > 12 mm [22, 23].

### 2.4 Outcome Evaluation

The study outcomes were 30‐day mortality and 6‐month functional outcome. Functional outcomes were assessed using the modified Rankin Scale (mRS), with favorable outcomes defined as mRS 0–3 and unfavorable outcomes as mRS 4–6.

### 2.5 Classification of IVH Topographical Features

In the development cohort, 11 distinct IVH topographical patterns were defined based on the presence or absence of hemorrhage in four ventricular compartments: the left lateral ventricle, right lateral ventricle, third ventricle, and fourth ventricle. The patterns are as follows: (1) unilateral lateral ventricle only (left or right); (2) bilateral lateral ventricles only; (3) unilateral lateral + third ventricle; (4) unilateral lateral + fourth ventricle; (5) unilateral lateral + third + fourth ventricles; (6) bilateral lateral + third ventricle; (7) bilateral lateral + fourth ventricle; (8) bilateral lateral + third + fourth ventricles; (9) isolated third ventricle; (10) isolated fourth ventricle; (11) isolated third and fourth ventricles. Two senior neurosurgeons (R.Z. and J.L.), blinded to clinical outcomes, independently reviewed all CT scans to classify IVH topographical patterns. Inter‐rater reliability was assessed using Cohen's kappa coefficient, with values > 0.80 (on a scale from 0 to 1) considered to indicate excellent agreement. Discrepancies were resolved by consensus with a third senior neurosurgeon (A.C.).

### 2.6 IVH Topographical Score Development

First, we constructed three multivariate prognostic models: (1) baseline clinical factors + oGS, (2) baseline clinical factors + mGS, and (3) baseline clinical factors + IVH topographical features. Model discrimination was compared using the area under the receiver operating characteristic (ROC) curve (AUC) to assess whether IVH topographical features could substitute for two previous IVH scores in the prediction models.

Based on the regression coefficients (*β* values) of significant predictors from the constructed IVH topographical features model, a novel IVH scoring system named the IVH topographical score (ITS) was developed by assigning weighted points to these predictors. ITS reliability was validated in the external cohort (*n* = 102).

The ITS was compared with four established ICH scoring systems: the original ICH score (oICH) [10], ICH‐GS [11], and two modified ICH scores (mICH‐A and mICH‐B) [12]. Predictive performance was evaluated using ROC analysis, wherein curves were plotted, AUCs were calculated, and the optimal diagnostic cutoff values for each scoring system were determined. Confusion matrices were constructed, and the accuracy, sensitivity, specificity, precision, and F1‐score were determined.

### 2.7 Statistical Analysis

All statistical analyses were performed using R software (version 2020; R Foundation for Statistical Computing; Vienna, Austria). Statistical significance was set at *p* < 0.05. Categorical variables are expressed as frequencies (percentages) and compared using the chi‐square test or Fisher's exact test. Continuous variables with a normal distribution are presented as mean ± standard deviation and compared using the independent sample *t*‐test. Non‐normally distributed continuous variables are presented as median (interquartile range) and compared using the Mann–Whitney *U* test. Potential confounding factors were adjusted for in multivariable logistic regression models to identify independent predictors of mortality. As this was a retrospective analysis of a prospectively maintained database, no formal sample size calculation was performed prior to the study. All eligible patients meeting the inclusion criteria during the study period were included. A post hoc power analysis indicated that with 385 patients and 91 events, the study had 80% power to detect an odds ratio of 1.8 for a predictor with a prevalence of 20% at an alpha level of 0.05.

## 3 Results

### 3.1 Internal Cohort

Table 1 summarizes the univariate analysis and multivariate logistic regression analyses in the internal cohort. Among the 385 patients, 294 (76.4%) survived and 91 (23.6%) died within 30 days. The mean age was similar between the two groups (64.6 ± 12.1 years vs. 66.2 ± 12.0 years, respectively). The mortality group had significantly lower Glasgow Coma Scale (GCS) scores on admission (6.0 ± 2.6 vs. 11.0 ± 3.6, *p* < 0.001), higher oGS (7.6 ± 2.9 vs. 4.2 ± 2.4, *p* < 0.001), higher mGS (17.2 ± 7.9 vs. 8.2 ± 6.4, *p* < 0.001), larger hematoma volumes (20.8 ± 12.1 vs. 10.6 ± 8.9, *p* < 0.001), and a higher likelihood of hydrocephalus (63.7% vs. 9.8%, *p* < 0.001) than the survival group. No significant differences were observed between the two groups in terms of hematoma location. Although the mortality group showed higher rates of hematoma evacuation (25.3% vs. 18.7%, *p* = 0.175) and EVD placement (40.6% vs. 33.7%, *p* = 0.224), these differences were not statistically significant. The mortality group exhibited a significantly lower proportion of isolated lateral ventricular involvement (unilateral: 6.6% vs. 38.1%, *p* < 0.001; bilateral: 2.2% vs. 10.2%, *p* = 0.029) and a significantly higher proportion of panventricular involvement (62.6% vs. 24.1%, *p* < 0.001). For the classification of IVH topographical patterns, inter‐rater agreement was excellent, with a Cohen's kappa of 0.89 (95% CI: 0.84–0.94).

**TABLE 1:** Univariate and multivariate logistic regression analysis of factors associated with 30‐day mortality in IVH patients from the internal cohort.

Variables	Survivors (*n* = 294)	Death (*n* = 91)	Univariate analysis OR (95% CI)	*p*	Multivariate analysis OR (95% CI)	*p*
Age	64.6 ± 12.1	66.2 ± 12.0	1.01 (0.99–1.03)	0.292		
Male	197 (67.0%)	64 (70.3%)	1.17 (0.70–1.95)	0.554		
Hypertension	237 (80.6%)	72 (79.1%)	0.91 (0.51–1.63)	0.755		
Diabetes	36 (12.2%)	13 (14.3%)	1.19 (0.60–2.36)	0.610		
History of stroke	28 (9.5%)	10 (10.9%)	1.17 (0.55–2.52)	0.682		
Coronary heart disease	39 (13.3%)	13 (14.3%)	1.09 (0.55–2.14)	0.804		
Temperature, °C	36.5 ± 0.5	36.5 ± 0.3	1.09 (0.55–2.14)	0.513		
Systolic BP, mmHg	172.4 ± 21.5	161.7 ± 23.3	1.00 (0.99–1.01)	0.823		
Diastolic BP, mmHg	87.7 ± 11.7	86.6 ± 15.5	0.99 (0.98–1.01)	0.435		
GCS score	11.0 ± 3.6	6.0 ± 2.6	0.64 (0.58–0.71)	< 0.001***	0.77 (0.69–0.87)	< 0.001***
Graeb score	4.2 ± 2.4	7.6 ± 2.9	1.53 (1.39–1.68)	< 0.001***		
Modified Graeb score	8.2 ± 6.4	17.2 ± 7.9	1.17 (1.13–1.21)	< 0.001***		
Hematoma volume, mL	10.6 ± 8.9	20.8 ± 12.1	1.09 (1.06–1.12)	< 0.001***	1.07 (1.06–1.12)	< 0.001***
Hydrocephalus	29 (9.8%)	58 (63.7%)	16.06 (9.05–28.52)	< 0.001***	4.02 (1.85–8.72)	< 0.001***
IVH pattern						
Unilat only	112 (38.1%)	6 (6.6%)	0.11 (0.05–0.27)	< 0.001***	0.39 (0.24–1.97)	0.485
Bilat only	30 (10.2%)	2 (2.2%)	0.20 (0.05–0.84)	0.029*		
Unilat + 3rd	20 (6.8%)	3 (3.3%)	0.57 (0.14–1.61)	0.228		
Unilat + 4th	4 (1.4%)	1 (1.1%)	0.81 (0.09–7.30)	1.000		
Unilat + 3rd + 4th	20 (6.8%)	5 (5.5%)	0.80 (0.29–2.18)	0.810		
Bilat +3 rd	17 (5.7%)	7 (7.7%)	1.36 (0.54–3.38)	0.468		
Bilat + 4th	2 (0.7%)	1 (1.1%)	1.62 (0.15–18.10)	0.694		
Bilat + 3rd + 4th	71 (24.1%)	57 (62.6%)	5.27 (3.19–8.71)	< 0.001***	3.16 (0.96–4.85)	0.063
3rd only	3 (1.0%)	1 (1.1%)	1.08 (0.11–10.50)	1.000		
4th only	3 (1.0%)	1 (1.1%)	1.08 (0.11–10.50)	1.000		
3rd + 4th only	12 (4.1%)	7 (7.7%)	1.96 (0.75–5.13)	0.172		
Localization						
Thalamus	153 (52.0%)	38 (42.9%)	0.66 (0.41–1.06)	0.088		
Basal ganglia	56 (19.0%)	20 (21.9%)	1.20 (0.67–2.13)	0.540		
Cerebellum	32 (10.9%)	17 (18.7%)	1.88 (0.99–3.58)	0.054		
Lobar	17 (5.8%)	6 (6.6%)	1.15 (0.44–3.01)	0.776		
Primary IVH	32 (10.9%)	9 (9.9%)	0.90 (0.41–1.96)	0.788		
Treatment						
Hematoma evacuation	55 (18.7%)	23 (25.3%)	1.47 (0.84–2.56)	0.175		
EVD	99 (33.7%)	37 (40.6%)	1.35 (0.83–2.19)	0.224		

Abbreviations: 3rd, third ventricle; 4th, fourth ventricle; Bilat, bilateral; CI, confidence interval; EVD, external ventricular drainage; GCS, Glasgow Coma Scale; ICH, intracerebral hemorrhage; IVH, intraventricular hemorrhage; OR, odds ratio; Unilat, unilateral.

* *p* < 0.05.

** *p* < 0.01.

*** *p* < 0.001.

### 3.2 Predictors of 30‐Day Mortality

The overall 30‐day mortality rate in the internal cohort was 23.6%. Comparative analysis of the predictive models showed that the IVH topographical features significantly outperformed the oGS and mGS in predicting mortality (AUC: 0.941 vs. 0.807 and 0.806, respectively; all *p* < 0.05, DeLong test; Figure 2A). Multivariate logistic regression identified IVH topographical features (odds ratio [OR]: 3.16; 95% confidence interval [CI]: 0.96–4.85; *p* = 0.033), hydrocephalus (OR: 4.02; 95% CI: 1.85–8.72; *p* < 0.001), hematoma volume (OR: 1.07; 95% CI: 1.04–1.10; *p* < 0.001), and GCS score (OR: 0.77; 95% CI: 0.69–0.87; *p* < 0.001) as independent predictors of 30‐day mortality (Table 1).

### 3.3 Role of IVH Topographical Patterns

**FIGURE 1:**
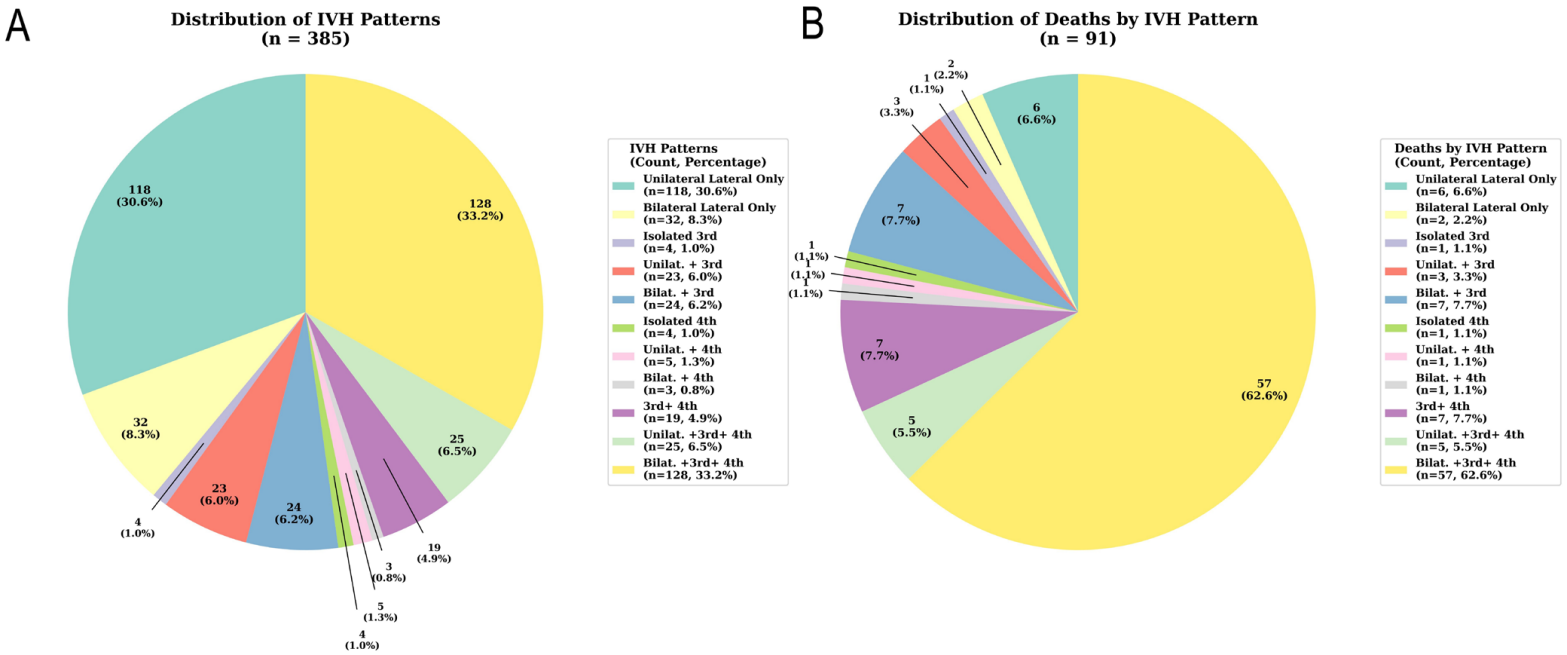
Spatial distribution patterns of intraventricular hemorrhage (IVH) and associated mortality. (A) Overall spatial distribution patterns of IVH occurrence in the internal cohort (*N* = 385). (B) Distribution of 30‐day mortality across IVH spatial classification patterns.

All observed topographical patterns of IVH occurrence and mortality rates are shown in Figure 1. Unilateral lateral ventricular hemorrhage was one of the most common patterns (*n* = 118; 30.6%) but was associated with a relatively low mortality rate (5.0%). In contrast, patterns involving the third and/or fourth ventricles were associated with substantially higher mortality rates, particularly bilateral lateral hemorrhage with third and fourth ventricular involvement (*n* = 128; 33.2%; mortality, 44.5%) and isolated third and fourth ventricular hemorrhage (*n* = 19; 4.9%; mortality, 36.8%). Isolated third or fourth ventricular hemorrhage had a mortality rate of 25.0%. Furthermore, bilateral lateral hemorrhage with third or fourth ventricular involvement was associated with a significantly higher risk of death than unilateral lateral hemorrhage with similar ventricular involvement. Detailed frequencies and mortality rates for all 11 patterns are provided in Figure 1.

### 3.4 ITS Development

The ITS was developed based on the independent predictors identified by the logistic regression analysis (Table 1). Each predictor was assigned a different point value, yielding a possible total score of 9 (Table 2). The 11 IVH topographical patterns were grouped according to their ORs and assigned points as follows: isolated lateral ventricular hemorrhage (including unilateral and bilateral), which had the lowest OR, was assigned 0 points; isolated third or fourth ventricular hemorrhage, as well as unilateral lateral hemorrhage with third or fourth ventricular involvement, all of which showed similar ORs, were assigned 1 point; combined third and fourth ventricular hemorrhage and bilateral lateral hemorrhage with third or fourth ventricular involvement, both of which had approximately double the OR of the previous group, were assigned 2 points; and panventricular hemorrhage (bilateral lateral hemorrhage with both third and fourth ventricular involvement) was assigned 3 points. GCS scores were categorized as 3–8 and 9–15 points and assigned 1 and 0 points, respectively. Hematoma volume was dichotomized as ≥ 20 mL or < 20 mL and assigned 1 or 0 points, respectively. Hydrocephalus was treated as a binary variable and assigned 4 or 0 points, respectively.

**TABLE 2:** The IVH topographical score (ITS) scoring system.

Component	Score
IVH pattern	
Unilat only/Bilat only	0
Unilat + 3rd/Unilat + 4th/Unilat + 3rd + 4th/3rd only/4th only	1
Bilat +3rd/Bilat + 4th/3rd + 4th only	2
Bilat + 3rd + 4th	3
GCS score	
3–8	1
9–15	0
ICH volume	
< 20 mL	0
≥ 20 mL	1
Hydrocephalus	
No	0
Yes	4

Abbreviations: 3rd, third ventricle; 4th, fourth ventricle; Bilat, bilateral; GCS, Glasgow Coma Scale; ICH, intracerebral hemorrhage; IVH, intraventricular hemorrhage; Unilat, unilateral.

The relationship between increasing ITS and 30‐day mortality is shown in Figure 3A. Among patients with scores of 0, 1, 2, 3, 4, 5, 6, 7, 8, and 9, the corresponding 30‐day mortality rates were 0.9%, 7.0%, 8.3%, 19.6%, 39.3%, 40.0%, 50.2%, 55.5%, 66.7%, and 90.6%, respectively.

### 3.5 Score Comparison

**FIGURE 2:**
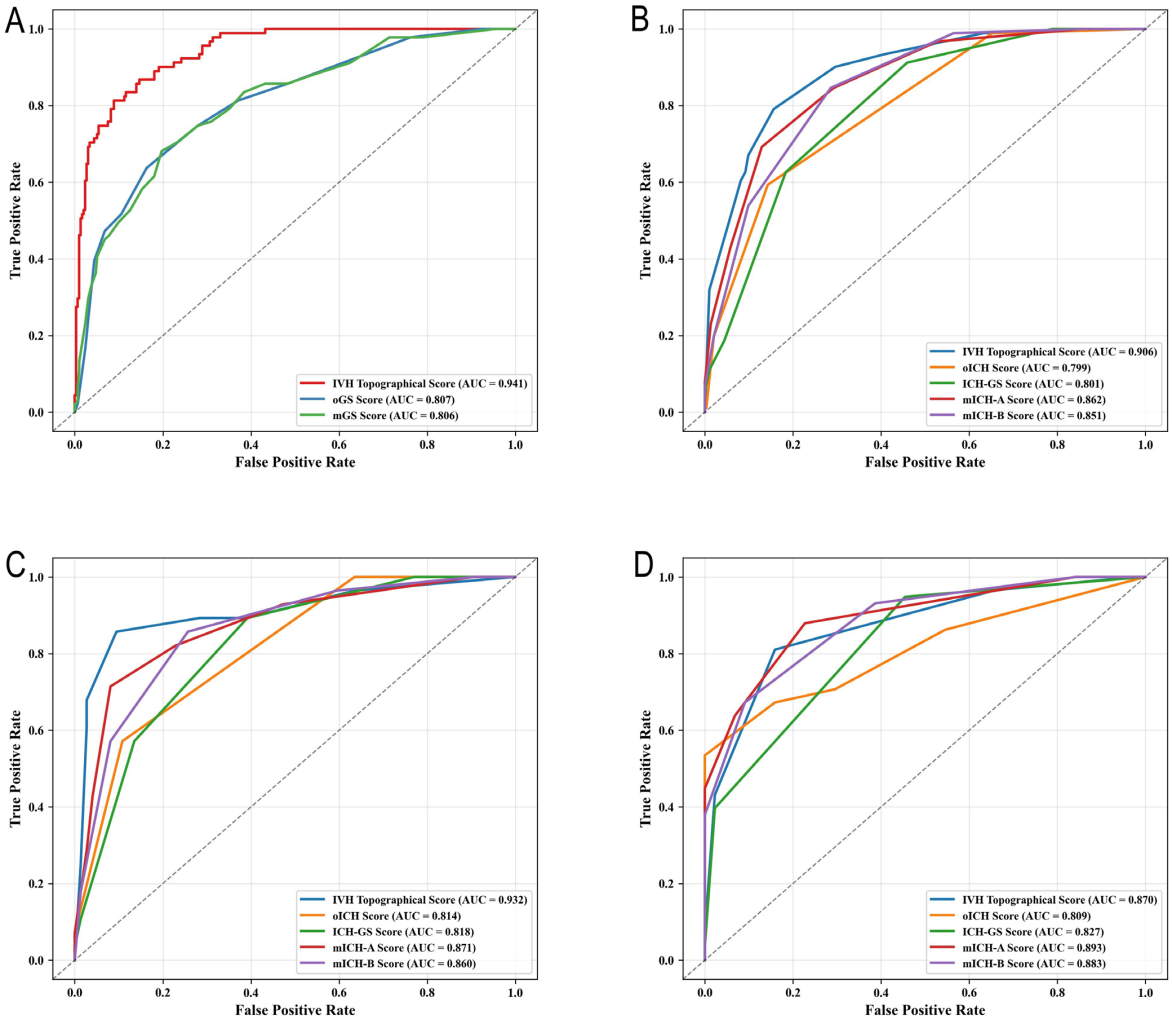
Comparative performance of IVH‐related metrics and scoring systems. (A) Comparison of IVH spatial features, original Graeb score (oGS), and modified Graeb score (mGS) in predicting 30‐day mortality in the internal cohort. (B) Comparison of the ISS with four previously established general ICH scores for predicting 30‐day mortality in the internal cohort. (C, D) Comparison of the ISS with the four general ICH scores for predicting 30‐day mortality (C) and 6‐month unfavorable outcomes (D) in the external validation cohort.

In the internal cohort, the ITS showed the highest AUC (0.906) for predicting 30‐day mortality (all *p* < 0.05, DeLong test), followed by the mICH‐A (0.862), mICH‐B (0.851), ICH‐GS (0.801), and oICH (0.799) scores (Figure 2B). In the external cohort, the ITS also showed the highest AUC (0.932) (all *p* < 0.05, DeLong test), followed by the mICH‐A (0.871), mICH‐B (0.860), ICH‐GS (0.818), and oICH (0.814) scores (Figure 2C). The mICH‐A score achieved the highest AUC (0.893) for predicting 6‐month functional outcomes (Figure 2D), followed by the ITS (0.887), mICH‐B (0.883), ICH‐GS (0.823), and oICH (0.809) scores. Calibration and decision curve analyses are provided in the Figures S1 and S2, respectively.

At the optimal cut‐off value determined by maximizing Youden's index in the internal cohort, confusion matrices were constructed for each scoring system to evaluate their classification performance for 30‐day mortality. The ITS demonstrated the highest overall accuracy (0.844), sensitivity (0.604), and F1‐score (0.647), indicating superior performance in identifying patients at risk of death while maintaining a balanced trade‐off with precision (0.696) and specificity (0.918). The confusion matrices and detailed comparative metrics for all scores are provided in the Figure S3 and Table S1, respectively.

### 3.6 External Cohort

Among the 102 patients in the external cohort (mean age 65.2 ± 12.3 years; range 33–89), 28 died (30‐day mortality rate: 27.5%). At 6 months, 42 (41.1%) patients achieved good functional recovery (mRS ≤ 3). Hemorrhage locations included the thalamus (50%), basal ganglia (16.7%), cerebellum (9.8%), lobar regions (6.8%), and primary IVH (11.8%). Detailed baseline characteristics of the external cohort stratified by outcome are presented in Table 3.

**TABLE 3:** Thirty‐day mortality and 6‐month outcomes in the external validation cohort.

Variables	Death (*n* = 28)	Survival (*n* = 74)	*p*	Unfavorable outcome (*n* = 60)	Favorable outcome (*n* = 42)	*p*
Age	63.4 ± 13.5	65.8 ± 11.9	0.376	65.4 ± 12.0	64.9 ± 12.9	0.851
Male	18 (64.3%)	57 (77.0%)	0.293	47 (78.3%)	28 (66.7%)	0.277
Hypertension	21 (75.0%)	56 (75.7%)	1.000	47 (78.3%)	30 (71.4%)	0.572
Diabetes	1 (3.6%)	9 (12.2%)	0.277	7 (11.7%)	3 (7.1%)	0.518
History of stroke	3 (10.7%)	7 (9.5%)	1.000	5 (8.3%)	5 (11.9%)	0.795
Coronary heart disease	4 (14.3%)	7 (9.5%)	0.488	6 (10.0%)	5 (11.9%)	1.000
Temperature, °C	36.6 ± 0.2	36.6 ± 0.3	0.574	36.5 ± 0.3	36.6 ± 0.3	0.905
Systolic BP, mmHg	171.5 ± 37.5	170.0 ± 32.8	0.455	172.1 ± 36.6	167.9 ± 30.1	0.371
Diastolic BP, mmHg	93.2 ± 20.2	95.3 ± 17.2	0.821	95.1 ± 20.8	94.3 ± 13.2	0.815
GCS score	6.6 ± 2.6	12.0 ± 3	< 0.001***	8.3 ± 3.1	13.7 ± 1.7	< 0.001***
Graeb score	8.1 ± 2.7	4.0 ± 2.4	< 0.001***	6.53 ± 2.9	3.14 ± 1.9	< 0.001***
Modified Graeb score	18.1 ± 7.6	7.8 ± 5.9	< 0.001***	14.0 ± 7.9	5.8 ± 4.6	< 0.001***
Hematoma volume, mL	19.8 ± 12.7	8.4 ± 7.9	< 0.001***	15.5 ± 11.4	5.7 ± 6.1	< 0.001***
Hydrocephalus	18 (64.3%)	2 (2.7%)	< 0.001***	20 (33.3%)	0 (0.0%)	< 0.001***
IVH pattern						
Unilat only	3 (10.7%)	26 (35.1%)	0.022*	13 (21.7%)	16 (38.1%)	0.125
Bilat only	0 (0%)	6 (8.1%)	1.000	2 (5%)	4 (9.5%)	0.248
Unilat + 3rd	0 (0%)	3 (4.1%)	1.000	1 (1.7%)	2 (4.8%)	0.412
Unilat + 4th	0 (0%)	1 (1.4%)	1.000	0 (0%)	1 (2.4%)	1.000
Unilat + 3rd + 4th	0 (0%)	5 (6.8%)	1.000	2 (3.3%)	3 (7.1%)	0.443
Bilat + 3rd	1 (3.6%)	5 (6.8%)	0.549	3 (5%)	3 (7.1%)	0.727
Bilat + 4th	0 (0%)	1 (1.4%)	1.000	1 (1.7%)	0 (0%)	1.000
Bilat + 3rd + 4th	23 (82.1%)	19 (25.7%)	< 0.001***	35 (58.3%)	7 (1.7%)	< 0.001***
3rd only	0 (0%)	1 (1.4%)	1.000	0 (0%)	1 (2.4%)	1.000
4th only	0 (0%)	4 (5.4%)	1.000	0 (0%)	4 (9.5%)	1.000
3rd + 4th only	2 (7.1%)	3 (4.1%)	0.524	3 (5%)	2 (4.8%)	0.885
Localization						
Thalamus	10 (35.7%)	41 (55.4%)	0.120	28 (46.7%)	23 (54.8%)	0.546
Basal ganglia	6 (21.4%)	11 (14.9%)	0.619	14 (23.3%)	3 (7.1%)	0.034*
Cerebellum	4 (14.3%)	6 (8.1%)	0.455	6 (10%)	4 (9.5%)	1.000
Lobar	4 (14.3%)	3 (4.1%)	0.087	4 (6.7%)	3 (7.1%)	1.000
Primary IVH	3 (10.7%)	9 (12.2%)	1.000	5 (8.3%)	7 (16.7%)	0.330
Treatment						
Hematoma evacuation	9 (32.1%)	6 (8.1%)	0.006**	13 (21.7%)	2 (4.8%)	0.022 *
EVD	11 (39.3%)	14 (18.9%)	0.060	19 (31.7%)	6 (14.8%)	0.076

Abbreviations: 3rd, third ventricle; 4th, fourth ventricle; Bilat, bilateral; BP, blood pressure; EVD, external ventricular drainage; GCS, Glasgow Coma Scale; ICH, intracerebral hemorrhage; IVH, intraventricular hemorrhage; Unilat, unilateral.

* *p* < 0.05.

** *p* < 0.01.

*** *p* < 0.001.

### 3.7 Outcomes

**FIGURE 3:**
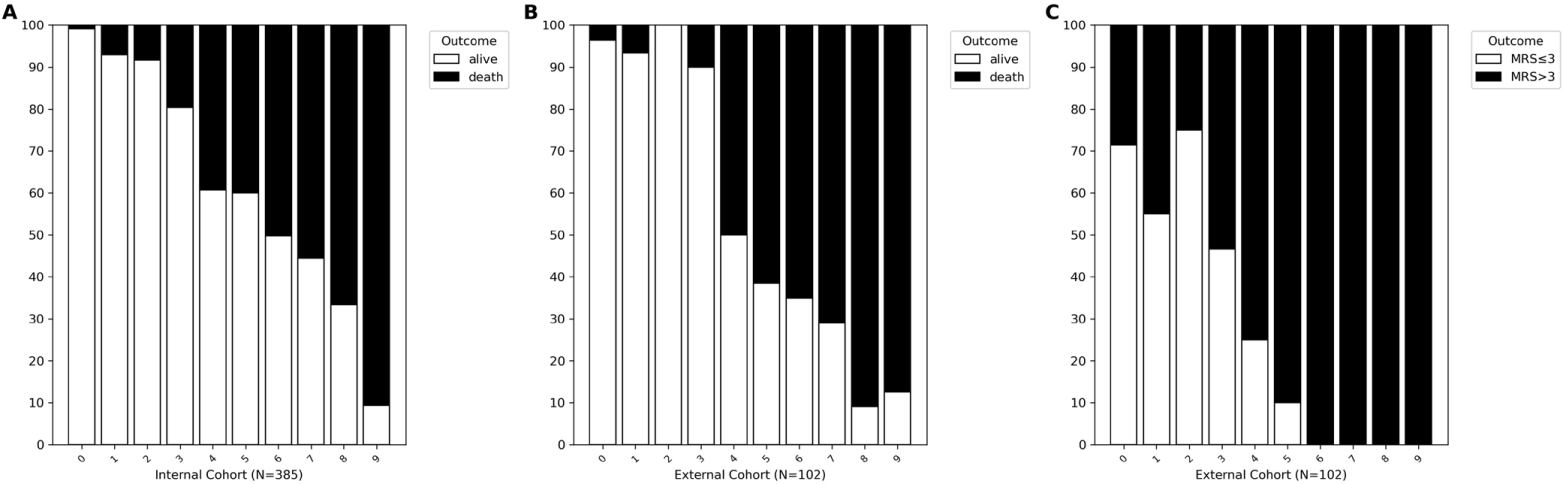
Association between ISS and clinical outcomes. (A) Relationship between ISS and 30‐day mortality in the internal cohort. Among patients with scores of 0, 1, 2, 3, 4, 5, 6, 7, 8, and 9, the corresponding 30‐day mortality rates were 0.9%, 7.0%, 8.3%, 19.6%, 39.3%, 40.0%, 50.2%, 55.5%, 66.7%, and 90.6%, respectively. (B) Relationship between ISS and 30‐day mortality in the external validation cohort. Among patients with scores of 0, 1, 2, 3, 4, 5, 6, 7, 8, and 9, the corresponding 30‐day mortality rates were 3.6%, 6.7%, 0%, 10.0%, 50.0%, 61.5%, 65.1%, 71.0%, 90.9%, and 87.5%, respectively. (C) Relationship between ISS and 6‐month functional outcomes in the external validation cohort. No patient with an ISS > 5 points achieved favorable functional recovery. Among patients with ISS scores of 0, 1, 2, 3, 4, and 5, the corresponding rates of unfavorable outcome were 28.6%, 45.0%, 25.0%, 53.3%, 75.0%, and 90.0%, respectively.

At 30‐day post‐ICH, among patients with scores of 0, 1, 2, 3, 4, 5, 6, 7, 8, and 9, the corresponding 30‐day mortality rates were 3.6%, 6.7%, 0%, 10.0%, 50.0%, 61.5%, 65.1%, 71.0%, 90.9%, and 87.5%, respectively (Figure 3B). At 6 months post‐ICH, unfavorable outcomes were observed across all ITS categories. No patient with an ITS > 5 points achieved favorable functional recovery. Among patients with ITS scores of 0, 1, 2, 3, 4, and 5, the rates of unfavorable outcomes were 28.6%, 45.0%, 25.0%, 53.3%, 75%, and 90%, respectively (Figure 3C).

Based on the relationship between the ITS and 30‐day mortality, we developed a risk stratification to classify patients into three risk groups: low risk (ITS 0–2), intermediate risk (ITS 3–6), and high risk (ITS 7–9). In the internal cohort, mortality rates were 5.2% (low), 35.8% (intermediate), and 72.4% (high). In the external validation cohort, the corresponding rates were 4.5%, 53.2%, and 86.5% (Table S2). This three‐tier stratification provides a practical tool for bedside risk assessment.

### 3.8 Subgroup and Sensitivity Analyses

We performed prespecified subgroup analyses stratified by age, sex, hematoma volume, GCS score, treatment modality, and hydrocephalus. The ITS maintained good discriminative ability for 6‐month functional outcome across all subgroups in the external cohort (Table S3). Several sensitivity analyses were conducted to assess the robustness of our findings. When a favorable functional outcome was redefined as mRS ≤ 2 (instead of mRS ≤ 3), the ITS remained predictive for 6‐month outcome (AUC 0.817, 95% CI 0.736–0.898) (Figure S4). When 6‐month mortality was used as an alternative endpoint, results were similar (AUC 0.893, 95% CI 0.827–0.959) (Figure S5). We also explored whether the effect of the 11‐category topographical classification was modified by hydrocephalus (Table S4). The interaction term was not statistically significant (OR = 0.24, 95% CI: 0.007–8.56, *p* = 0.43), indicating no detectable synergistic effect in our dataset. However, it cannot be excluded that this null finding is due to limited statistical power. Therefore, these findings should be interpreted with caution.

To assess the robustness of our classification against the modified Graeb score, we performed Spearman correlation analysis and pure imaging model comparisons. The classification was strongly correlated with the mGS (ρ = 0.847, *p* < 0.001). In pure imaging models without clinical covariates, the 11‐category topographical classification predicted 30‐day mortality with an AUC of 0.793 (95% CI: 0.75–0.84), which was comparable to that of the mGS (AUC 0.806, 95% CI: 0.76–0.85; DeLong test, *p* = 0.411). Detailed results are provided in Figures S6 and S7, respectively.

## 4 Discussion

This study systematically analyzed the association between the overall topographical distribution of IVH and patient prognosis. In a cohort of 385 patients with IVH and hematoma volume < 40 mL, IVH topographical patterns, hydrocephalus, hematoma volume, and GCS score were identified as independent predictors of 30‐day mortality. We further highlighted the role of IVH topographical patterns in these patients by introducing a four‐level grading system and developing a dedicated IVH scoring system, the ITS, which was evaluated for its ability to predict mortality and functional outcomes in both internal and external cohorts. Compared with several previously established general ICH scores [10, 11, 12], the ITS demonstrated superior performance for predicting 30‐day mortality in both cohorts, with comparable performance for predicting 6‐month functional outcomes in the external validation cohort.

Previous studies have identified IVH as a strong independent predictor of poor prognosis following ICH [5, 6, 7, 8, 9]. However, the influence of the overall topographical distribution of IVH within the ventricular system on prognosis has not been systematically elucidated. Existing clinical evidence has primarily focused on the isolated effects of specific ventricles. Hemorrhage with third or fourth ventricle involvement may be most closely associated with poor prognosis in patients with IVH, likely due to direct compression of, or damage to, the brainstem and surrounding critical structures, which are often irreversible [18, 19, 20]. In contrast, isolated lateral ventricular hemorrhage is associated with a relatively better prognosis, possibly owing to the “buffering” effect provided by the larger volume of the lateral ventricles and the potential for partial neurological recovery following IVH clearance [20]. However, previous studies have not clarified the differential risk between unilateral and bilateral lateral ventricular involvement. Additionally, the number of ventricles involved, total IVH volume, and secondary hydrocephalus have been shown to be closely related to prognosis in prior research [3, 4, 17].

This study provides a systematic characterization of the independent prognostic risks associated with different topographical patterns of IVH. Through classification of these patterns, we confirmed that third or fourth ventricle involvement significantly increased mortality risk compared with isolated lateral ventricular hemorrhage, with nearly similar risk magnitude observed for the third and fourth ventricle involvement. Furthermore, unilateral lateral hemorrhage with third or fourth ventricular involvement did not confer a significantly increased mortality risk compared with isolated third or fourth ventricular hemorrhage. This further underscores the independent and dominant role of the third and fourth ventricles in predicting prognosis in patients with IVH. Additionally, we found that bilateral lateral hemorrhage with third or fourth ventricular involvement was associated with a significantly higher mortality risk compared with unilateral lateral hemorrhage involving the same ventricles. Combined third and fourth ventricular hemorrhage conferred a risk comparable to that of bilateral lateral hemorrhage with third or fourth ventricular involvement, whereas isolated third or fourth ventricular hemorrhage alone did not reach such a level of risk.

Specifically, our findings revealed a clear progressive pattern of overall IVH risk: isolated lateral ventricular hemorrhage had the lowest risk coefficient, with no significant difference between unilateral and bilateral involvement. Isolated third or fourth ventricular involvement had risk coefficients similar to those of unilateral lateral hemorrhage with third or fourth ventricular involvement. Bilateral lateral hemorrhage with third or fourth ventricular involvement had risk coefficients comparable to those of combined third and fourth ventricular hemorrhage. Pantventricular hemorrhage carried the highest risk coefficient. Moreover, the risk coefficient approximately doubled with each successive level of this four‐tiered classification.

Accurate and rapid assessment of IVH is critical for clinical risk stratification, monitoring disease progression, and evaluating treatment efficacy. In the present study, we first confirmed that the prognostic model incorporating the 11‐category IVH topographical features significantly outperformed models containing the original Graeb score (oGS) or the modified Graeb score (mGS) in predicting 30‐day mortality in patients with IVH (AUC: 0.941 vs. 0.807 and 0.806, respectively). However, no statistically significant difference was observed between the oGS and mGS. The mGS was proposed by Morgan et al. with the aim of improving outcome prediction by incorporating detailed hemorrhage location within the ventricular system [17]. Despite its more refined design, several subsequent studies have suggested that oGS and mGS may perform comparably in terms of final predictive performance, with both exhibiting good interobserver reliability in the hyperacute phase [24, 25, 26]. Beyond its superior predictive performance compared with the two conventional IVH scores, a key advantage of the ITS is its ability to rapidly estimate overall IVH severity. It can be directly used to predict mortality without the need for time‐consuming calculations and better aligns with the practical demands of clinical settings. In contrast, existing IVH scores remain cumbersome and time‐consuming and are not specifically designed for predicting mortality or poor outcomes.

Previous ICH scoring systems have been developed for patients with ICH, regardless of the presence or absence of IVH [13]. Although the mICH‐A and mICH‐B scores incorporate assessments, such as the oGS, to characterize and quantify the extent of IVH, the other two scoring systems only treat IVH as a simple binary variable (present or absent). Comparison of the final model demonstrated that the ITS, which incorporates the overall hemorrhage pattern of IVH, exhibited a significantly better predictive performance than the four previous ICH scores [10, 11, 12]. In addition, we found that the predictive performance of the mICH‐A and mICH‐B scores was significantly higher than that of ICH‐GS and oICH, further indicating the strong predictive role of IVH extent in prognostic models for patients with ICH with concurrent IVH. These findings underscore the need for a dedicated IVH prognostic scoring system, rather than relying solely on traditional ICH scores, to assess outcomes in patients with IVH.

Furthermore, the comparable performance of these scores in predicting long‐term functional outcomes suggests that the long‐term neurological recovery of most patients with IVH is more strongly influenced by the extent of the primary parenchymal hemorrhage than by IVH severity. This finding has significant clinical implications and indicates that IVH‐related secondary injuries may be effectively mitigated or partially reversed through active interventions (such as EVD or pharmacological treatments).

Acute hydrocephalus occurs in approximately 40%–50% of patients with IVH and may present as a life‐threatening complication [2, 3, 5, 6]. Previous studies have established hydrocephalus as an independent risk factor for predicting 30‐day mortality in patients with ICH [2, 5, 6, 27]. The development of hydrocephalus results from both mechanical obstruction by blood clots and secondary inflammatory responses mediated by components such as hemoglobin and iron ions [3]. In this study, hydrocephalus was observed in 63.7% of patients who died within 30 days in the internal cohort and in 64.3% in the external cohort. As a clinical marker of the end‐stage of severe IVH, hydrocephalus was incorporated as the strongest predictor in our scoring system. In another study, the severity of hydrocephalus was used to develop a novel IVH scoring system; however, it lacked rigorous external validation and, most importantly, did not impose restrictions on parenchymal hematoma volume [2]. This may have, to some extent, obscured the true independent prognostic contribution of IVH. Although EVD is a rapid and life‐saving intervention for acute hydrocephalus, its optimal timing remains uncertain. One study indicated that involvement of ≥ 3 ventricles represented the highest‐risk feature for EVD requirement [28]. Consequently, the ITS can aid in identifying high‐risk patients and offer an objective rationale for early risk stratification.

Admission GCS score and hematoma volume are among the most established and consistent predictors of ICH prognosis, a finding that has been validated in nearly all prognostic models [10, 11, 12, 13]. Although the present study limited the parenchymal hematoma volume (< 40 mL) to highlight the independent role of IVH, GCS score and hematoma volume still demonstrated a strong independent predictive value in our IVH prediction model.

### 4.1 Limitations

Our study has several limitations. First, although we included a relatively large single‐center cohort of patients with IVH, the sample size of the development cohort remained limited owing to strict inclusion and exclusion criteria. In particular, the number of patients with isolated third ventricular or fourth ventricular hemorrhage was relatively small, which may have led to insufficient statistical power. Therefore, the negative findings for these patterns should be interpreted with caution. Second, both the internal and external validation cohorts in this study included patients who underwent surgical interventions such as hematoma evacuation or EVD. Although uniform eligibility criteria were applied in both cohorts and surgical decisions themselves may reflect disease severity, treatment‐related bias could still have been introduced. Third, to minimize the confounding effect of early mortality primarily driven by massive parenchymal hemorrhage or cerebral herniation on the prognostic effect of IVH itself, this study set an upper limit of 40 mL for the parenchymal hematoma volume in the included patients. The same criterion was applied in the external validation cohort, which is consistent with previous research [20]. This threshold allowed us to focus more specifically on the independent contribution of IVH but limits generalizability to patients with larger hematomas. Therefore, further validation in unselected ICH populations and in patients with larger hematoma volumes (> 40 mL) is warranted. Fourth, although the ITS outperforms conventional IVH scores in predictive performance and can rapidly estimate overall IVH severity without time‐consuming calculations, we acknowledge that no formal head‐to‐head comparison of completion time or ease of use has been performed against existing scoring systems. Future implementation studies are needed to directly compare the clinical efficiency of the ITS with these tools. Finally, despite rigorous external validation, the ITS still requires further validation in larger multicenter cohorts and across different ethnic populations to assess its generalizability.

## 5 Conclusion

In conclusion, our study demonstrated that topographical patterns of IVH provide an intuitive and useful tool for risk stratification in patients with IVH. The ITS may represent a practical alternative to conventional IVH scoring methods. However, further prospective validation in larger and more diverse populations is warranted.

## Author Contributions

Conception and design: Ao Chen. Acquisition of data: Ao Chen, Xun Chen, RenHui Zhou, and JianXian Li. Drafting the article: Ao Chen. Analysis and interpretation of data: Ao Chen, Xufan Yu, and JiaYing Peng. Critically revising the article: Ao Chen. Study supervision: RenHui Zhou and JianXian Li. All authors read and approved the final manuscript.

## Funding

The authors have nothing to report.

## Ethics Statement

This study was approved by the institutional review board of Yueyang People's Hospital (approval no. 2019‐015) and The Fourth People's Hospital of Changsha (approval no. 2024‐066). This study was conducted in accordance with the guidelines of the ethics committee and the 1964 Declaration of Helsinki and its later amendments.

## Consent

Written informed consent was waived due to the retrospective nature of the study.

## Conflicts of Interest

The authors declare no conflicts of interest.

## Supporting information


**Figure S1:** Calibration plot of the ITS for predicting 30‐day mortality in the internal cohort.
**Figure S2:** Decision curve analysis (DCA) of the ITS for predicting 30‐day mortality in the internal cohort.
**Figure S3:** Confusion matrices of each scoring system for predicting 30‐day mortality. The ITS demonstrated the highest overall accuracy (0.844), sensitivity (0.604), and F1‐score (0.647), indicating superior performance in identifying patients at risk of death while maintaining a balanced trade‐off between precision (0.696) and specificity (0.918).
**Figure S4:** ROC curves of the ITS for predicting 6‐month functional outcome (mRS 0–3 vs. mRS 0–2) in the external cohort.
**Figure S5:** Receiver operating characteristic (ROC) curves of the ITS for predicting 30‐day mortality (AUC = 0.932) and 6‐month mortality (AUC = 0.863) in the external cohort.
**Figure S7:** Comparison of pure imaging models: IVH topographical classification vs. mGS.
**Table S1:** Comparison of predictive performance between the IVH spatial score and four general ICH scores.
**Table S2:** Risk stratification and 30‐day mortality by ITS score.
**Table S3:** Subgroup analysis of ITS for predicting 6‐month functional outcome in the external cohort.
**Table S4:** Interaction analysis of IVH pattern and hydrocephalus.

## Data Availability

The data that support the findings of this study are available from the corresponding author upon reasonable request.
