# Supplementary material for: Topographical Patterns of Intraventricular Hemorrhage as a Novel Prognostic Imaging Biomarker in Intracerebral Hemorrhage
Source: CNS Neurosci Ther. 2026 Aug 10;32(8):e71071. doi: 10.1002/cns.71071 (PMC13458277; doi:10.1002/cns.71071)
Supplement: Supplementary file 1 [file CNS-32-e71071-s001.docx]

**Supplementary Figure S1.** Calibration plot of the ITS for predicting 30‑day mortality in the internal cohort.

**Supplementary Figure S2.** Decision curve analysis (DCA) of the ITS for predicting 30‑day mortality in the internal cohort.

**Supplementary Figure S3.** Confusion matrices of each scoring system for predicting 30‑day mortality. The ITS demonstrated the highest overall accuracy (0.844), sensitivity (0.604), and F1‑score (0.647), indicating superior performance in identifying patients at risk of death while maintaining a balanced trade‑off between precision (0.696) and specificity (0.918).

**Supplementary Figure S4.** ROC curves of the ITS for predicting 6‑month functional outcome (mRS 0–3 vs. mRS 0–2) in the external cohort.

**Supplementary Figure S5.** Receiver operating characteristic (ROC) curves of the ITS for predicting 30‑day mortality (AUC = 0.932) and 6‑month mortality (AUC = 0.863) in the external cohort.

**Supplementary Figure S6.** Spearman correlation between ITS risk grade and modified Graeb score (mGS).

**Supplementary Figure S7.** Comparison of pure imaging models: IVH topographical classification vs. mGS.

**Supplementary Table S1. Comparison of Predictive Performance Between the IVH Spatial Score and Four General ICH Scores.**

| Model | Accuracy | Sensitivity | Specificity | Precision | F1-Score | AUC |
| --- | --- | --- | --- | --- | --- | --- |
| ISS | 0.844 | 0.604 | 0.918 | 0.696 | 0.647 | 0.906 |
| oICH | 0.795 | 0.198 | 0.98 | 0.75 | 0.313 | 0.799 |
| ICH-GS | 0.774 | 0.187 | 0.956 | 0.567 | 0.281 | 0.801 |
| mICH-A | 0.821 | 0.429 | 0.942 | 0.696 | 0.531 | 0.862 |
| mICH-B | 0.816 | 0.538 | 0.901 | 0.628 | 0.58 | 0.851 |

**Supplementary Table S2. Risk stratification and 30‑day mortality by ITS score.**

| Risk group | ITS score | Internal cohort | External cohort |
| --- | --- | --- | --- |
| Low | 0–2 | 5.2% (8/153) | 4.5% (2/44) |
| Intermediate | 3–6 | 35.8% (39/109) | 53.2% (33/62) |
| High | 7–9 | 72.4% (42/58) | 86.5% (32/37) |

**Supplementary Table S3. Subgroup analysis of ITS for predicting 6‑month functional outcome in the external cohort.**

| Subgroup | Category | n (events) | OR (95% CI) | P value |
| --- | --- | --- | --- | --- |
| EVD | No | 77 (36) | 0.56 (0.40–0.78) | <0.001 |
|  | Yes | 25 (7) | 0.23 (0.07–0.79) | 0.019 |
| Surgical evacuation | No | 87 (41) | 0.53(0.39-0.74) | <0.001 |
|  | Yes | 15 (2) | 0.37(0.09-1.59) | 0.182 |
| Age | <60 years | 31 (14) | 0.31 (0.13–0.75) | 0.009 |
|  | ≥ 60 years | 71 (29) | 0.52 (0.35–0.78) | 0.001 |
| Sex | Female | 27 (14) | 0.47 (0.26–0.86) | 0.014 |
|  | Male | 75 (29) | 0.54 (0.37–0.77) | <0.001 |
| GCS score | ≥ 9 | 70 (43) | 0.66 (0.45–0.97) | 0.035 |
|  | < 9 | 32 (0) | - | - |
| Hydrocephalus | No | 82 (43) | 0.53(0.38-0.76) | <0.001 |
|  | Yes | 20 (0) | - | - |
| Hematoma volume | < 20 mL | 83(41) | 0.51 (0.37–0.71) | <0.001 |
|  | ≥ 20 mL | 19(2) | 0.55 (0.18–1.69) | 0.295 |

**Supplementary Table S4. Interaction analysis of IVH pattern and hydrocephalus.**

| Variable | β (Coefficient) | OR | 95% CI | P value |
| --- | --- | --- | --- | --- |
| IVH pattern | 1.14 | 3.12 | 1.20 – 8.13 | 0.020 |
| Hydrocephalus | 2.91 | 18.35 | 0.55 – 614.87 | 0.104 |
| Interaction term | -1.44 | 0.24 | 0.007 – 8.56 | 0.431 |
| GCS score | -0.27 | 0.76 | 0.68 – 0.85 | <0.001 |
| Hematoma volume | 0.06 | 1.06 | 1.03 – 1.09 | <0.001 |
